# Investigation of the regulatory effects of tea polyphenols on CYP450s in HepG2 cells

**DOI:** 10.3389/fnut.2025.1663800

**Published:** 2025-10-09

**Authors:** Dan Zuo, Hong Ren, Zhaoxu Ren, Jieyu Chen, Feiyang Wang, Zixin Zhang, Haiyan Sun

**Affiliations:** ^1^Anshun Technical College, Anshun, China; ^2^School of Food and Drug, Shenzhen Polytechnic University, Shenzhen, China; ^3^Chongqing Business Vocational College, Chongqing, China

**Keywords:** tea polyphenols, CYP450s, food-drug interaction, mRNA, protein expression

## Abstract

**Introduction:**

Tea, one of the world's three major beverages, exhibits antioxidant, antitumour, and cardiovascular benefits, primarily due to its polyphenolic components. However, the roles of tea polyphenols on the modulation of cytochrome P450 enzymes (CYP450s) are not well documented. Therefore, this study investigates the regulatory effects of tea polyphenols on CYP450s in HepG2 cells.

**Methods:**

High-performance liquid chromatography (HPLC) was used to analyse the compositions of tea polyphenol extracts from Longjing green tea (unfermented), Tieguanyin oolong tea (semifermented) and Dianhong black tea (fully fermented). HepG2 cells were treated with these extracts and their major polyphenolic constituents (EGCG, EGC, ECG, TF, TF-3-G, and TF-3′-G), and the mRNA and protein expression levels of CYP3A4, CYP2E1, CYP2C9 and CYP1A2 were measured using real-time RT–PCR and Western blotting.

**Results:**

Significant regulation of CYP450 mRNA and protein expression by the three tea polyphenol extracts was observed, and enzyme inhibition was more prevalent than induction, with large contributions from the major monomers, including EGCG, EGC, and ECG. These findings indicate that interactions based on metabolism might occur when tea polyphenols are combined with medications.

**Conclusion:**

This study provides evidence that tea polyphenols significantly affect CYP450 enzyme expression, offering insights into the potential interactions between tea consumption and drug metabolism.

## 1 Introduction

Tea, which is widely consumed worldwide, provides significant health benefits because of its high content of tea polyphenols (15%−30% dry weight), including epigallocatechin gallate (EGCG), epigallocatechin (EGC), epicatechin gallate (ECG), theaflavin (TF), theaflavin-3-gallate (TF-3-G) and theaflavin-3′-digallate (TF-3′-G) (1). Extensive preclinical research indicates that these polyphenolic compounds (particularly EGCG) exhibit broad biological and pharmacological properties, including antioxidant, antitumor, and cardiovascular protective effects (2, 3). EGCG can improve cardiovascular health in obese subjects by directly acting as a sympathetic potentiator or by reducing the compensatory response after lowering blood pressure through its primary vasodilatory effect (4). Furthermore, Yin et al. (5) found that cancer patients tolerated inhalation of EGCG solutions via aerosol delivery systems well, suggesting EGCG may offer therapeutic potential for COVID-19-induced pneumonia.

Based on the fermentation level, teas can be divided into green teas, such as Longjing tea; oolong teas, such as Tieguanyin tea; and black teas, such as Dianhong tea (6). The Dianhong black tea used in this experiment was harvested from fresh, tender buds of large-leaf tea plants in Lincang City, Yunnan Province. The Tieguanyin tea plants belong to the Tieguanyin cultivar, also known as Red Heart Guanyin. The Longjing green tea was produced from tender buds and leaves of the Longjing No. 43 tea plant cultivar grown in Hangzhou's West Lake District. Both tea plants are clonal, shrub-type medium-leaf cultivars. Polyphenol content is influenced by multiple factors, including genetic traits, tea cultivar, climate, altitude, soil and fertilizer management, harvest season, leaf age, and processing techniques (7, 8). Among these, weather, season, and soil have been confirmed as the primary influencing factors (9). When daily temperatures rise, the levels of EGC, epicatechin (EC), and EGCG in tea leaves increase together, a process mediated by hormone signal transduction factors (10, 11). Soil biochemical properties not only determine the availability of essential nutrients, which are critical for plant growth, but also form the basis for the biosynthesis of chemical compounds (12). For example, Ran et al. (13) reported that, in paddy soil, the relative content of EGCG, ECG, and catechin (C) was significantly higher than in loess and red soil. Interestingly, the catechin content initially decreased and then increased with altitude.

Cytochrome P450 enzymes (CYP450s) are essential for the metabolism of drugs and other foreign substances. Previous studies have indicated that CYP450s are involved in the metabolism of more than 90% of clinical drugs (14). In addition to genetic factors, extrinsic factors, such as food, can affect CYP450 activity and/or expression. Previous studies have shown that green tea polyphenols can simultaneously inhibit the activity of CYP2B6, CYP2C8, CYP2C19, CYP2D6, and CYP3A (15). Another study (16) found that four theaflavins (theaflavin3'-gallate, theaflavin-3,3'-digallate, theaflavin-3-gallate, and theaflavin) all inhibit the expression of CYP1A2 and CYP2C8. The inhibition or induction of CYP450 enzyme activity, especially CYP1A2, CYP2C9, CYP2E1, and CYP3A4, is a major target for drug metabolic interactions, as their altered expression and quantity can lead to changes in the pharmacokinetic and even pharmacodynamic properties of the drug (17, 18).

According to data from the International Tea Committee, global tea consumption has been growing year by year, with global tea production reaching 7.052 million tons in 2024 (19). However, a thorough and systematic investigation into the impact of tea on medicines is lacking in the literature. Existing studies on tea polyphenols from around the world have focused primarily on the biological functions of tea, including its antioxidant, antineoplastic, and antibacterial properties (20, 21). Conversely, the influence of tea polyphenols on CYP450 enzymes has been inadequately explored, and the majority of related research has focused on CYP3A enzymes and utilized animal models (22). Therefore, this research aimed to evaluate whether tea polyphenols affect CYP450 gene expression in a HepG2 cell model, addressing the traditional belief that tea may interfere with drug efficacy.

Real-time RT–PCR and Western blotting were used to investigate the possible regulatory mechanisms of polyphenol extracts from Longjing green tea, Tieguanyin oolong tea and Dianhong black tea and their major monomers, such as catechins (EGCG, EGC, and ECG) and theaflavins (TF, TF-3-G, and TF-3′-G), on CYP450 isoforms (CYP3A4, CYP2E1, CYP2C9, and CYP1A2), at both the gene transcription and macroscopic protein expression levels. The goal of this study was also to assess possible tea-drug interactions and provide information on the pharmacology, toxicology, and adverse effects of tea consumption to promote the appropriate use of pharmaceuticals.

## 2 Materials and methods

### 2.1 Chemicals and reagents

Longjing green tea, Tieguanyin oolong tea and Dianhong black tea were purchased from Yipinxuan Teahouse (Shenzhen, Guangdong, China) and authenticated by Associate Professor Zhang Chunhui of the School of Food and Drug at Shenzhen Polytechnic University. The gallocatechin (GC), caffeine (CAF), gallic acid (GA), catechin gallate (CG), EGC, EC, C, EGCG, ECG, TF, gallocatechin gallate (GCG), TF-3-G and TF-3′-G standards were all chromatographic grade and purchased from Sigma-Aldrich (St. Louis, MO, USA). Dipotassium hydrogen phosphate (AR), potassium phosphate monobasic (AR) 4-(2-hydroxyethyl)-1-piperazineethanesulfonic acid (HEPES), dimethyl sulfoxide (DMSO), penicillin-streptomycin solution (100×) and Trypan blue were purchased from Sigma-Aldrich too (St. Louis, MO, USA). Trypsin-EDTA solution (0.05%) was purchased from Corning Incorporated (NY, USA). All chemicals are of the highest purity grade available.

### 2.2 Extraction of tea polyphenols

Following the Chinese national standard GB/T8313-2018 (23), tea polyphenols were extracted from Dianhong tea, Longjing green tea, and Tie Guanyin tea, with slight adjustments for laboratory conditions. Tea powder was mixed with 70% methanol at a ratio of 1:25 (m/V) and then subjected to condensation reflux extraction for 30 min; extraction was repeated 3 times. The filtrates were combined and concentrated by rotary evaporation to 15%−20% of the original volume, ensuring complete removal of the methanol. The filtrate was extracted with 3 times the volume of chloroform, and the extract was concentrated by rotary evaporation to ~5% of the original volume and dissolved in ultrapure water. The mixture was centrifuged at 10,000 r/min for 15 min, and the supernatant was vacuum freeze-dried for storage in a dark drying oven at room temperature.

### 2.3 Identification of tea polyphenols by high-performance liquid chromatography (HPLC)

The primary tea polyphenol components were identified using an LC-20A Shimadzu HPLC system (Shimadzu Technologies, Kyoto, Japan) with an Agilent ZORBAX Eclipse Plus Phenyl-Hexyl column (5 μm, 4.6 mm × 250 mm; Agilent Technologies, CA, USA) as the analytical column; SPD-M20A Detector. The mobile phase was composed of 0.17% (v/v) acetic acid in water (A) and 100% acetonitrile (B). Elution was performed at a flow rate was 1.0 ml/min, and the detection wavelength was 280 nm. Gradient elution was performed as follows: 0–10 min, 2%−3% B; 10–15 min, 3%−7% B; 15–30 min, 7%−10% B; 30–55 min, 10%−12% B; 55–65 min, 12%−14% B; 65–75 min, 14%−15% B; 75–78 min, 15%−25% B; 78–90 min, 25%−30% B; and 90–100 min, 2% B. The temperature of the column was maintained at 35 °C, and the injection volume for each sample was 10 μl.

### 2.4 Cell culture and treatment

HepG2 cells were obtained from the American Type Culture Collection (Bethesda, MD, USA) and cultured and maintained according to the manufacturer's guidelines. Briefly, HepG2 cells were cultured and maintained in Dulbecco's modified Eagle's medium (Gibco BRL, Paisley, UK) supplemented with 10% fetal bovine serum (Gibco BRL, Paisley, UK) and 1% antibiotics and antimycotics in 5% CO_2_ at 37 °C. HepG2 cells were subjected to tea polyphenol treatment and incubated with samples in the cytotoxic concentration range. In the tea polyphenol extract groups, the cells were incubated with different concentrations (100, 200 and 400 μg/ml) of tea polyphenol extracts for 24 h. In the tea polyphenol monomer groups, the cells were cultured with three different concentrations (20, 40 and 80 μg/ml) of tea polyphenol monomers for 24 h.

### 2.5 RNA isolation and real-time reverse transcription PCR

Total RNA was isolated from HepG2 cells treated with tea polyphenols using TRIzol reagent (Invitrogen, CA, USA) in accordance with the manufacturer's guidelines. The absorbance of each sample at 260 and 280 nm was measured by ultraviolet spectrophotometry. Samples with A260 nm/A280 nm values between 1.8 and 2.1 were utilized for real-time quantitative PCR (RT–PCR) analysis. Using a High-Capacity cDNA Archive Kit (Takara), 2 μg of each RNA sample was reverse transcribed. Real-time PCR was performed on a Light Cycler 2.0 Real-Time Detection System (Roche, CA, USA) with the SYBR^®^ Premix Ex Taq^TM^ kit (Takara). The cycling conditions were as follows: predenaturation at 95 °C for 30 s, denaturation at 95 °C for 5 s, annealing at 60 °C for 20 s, and extension at 65 °C for 15 s, for a total of 50 cycles. Sangon Biotech (Shanghai, China) synthesized all the primers, and their sequences are provided in Table 1. The mRNA expression levels of CYP450s were calculated using the 2^(−ΔΔCt)^ method after normalization to the level of β*-actin* expression.

**Table 1:** Primer sequences used for real-time polymerase chain reaction.

**Genes**	**Primer sequence (5** ^**′**^ **-3** ^**′**^ **)**		**Cycle**
CYP3A4	Forward primer	CAGGAGGAAATTGATGCAGTTTT	50
	Reverse primer	GTCAAGATACTCCATCTGTAGCACAGT	
CYP2C9	Forward primer	CTTGACACCACTCCAGTTGTC	50
	Reverse primer	AGATGGATAATGCCCCAGAG	
CYP2E1	Forward primer	GCAAGAGATGCCCTACATGGA	50
	Reverse primer	GGGCACGAGGGTGATGAA	
CYP1A2	Forward primer	AGCTTCTCCTGGCCTCTGC	50
	Reverse primer	GGACTTTTCAGGCCTTTGGG	
GAPDH	Forward primer	GGACCACCAGCCCCAGCAAGAG	50
	Reverse primer	GAGGAGGGGAGATTCAGTGTGGTG	

### 2.6 Protein isolation and western blotting

Protein expression in HepG2 cells was analyzed by Western blotting. Briefly, HepG2 cells were washed with cold PBS, placed in NP-40 lysate containing protease inhibitors for 20 min, and then centrifuged at 12,000 r/min for 30 min, after which the supernatant (whole-protein extract) was transferred to prefrozen centrifuge tubes. A BCA protein assay kit (Pierce, Rockford, IL, USA) was used for protein detection. Proteins were separated via 10% SDS–PAGE and then transferred onto a PVDF membrane via electrophoresis. The membrane was probed with anti-CYP3A4, anti-CYP1A2, anti-CYP2C9, anti-CYP2E1 and anti-GAPDH antibodies (Abcam, Cambridge, MA, USA). An X-ray film was used for chemiluminescent detection with an enhanced chemiluminescence substrate (Pierce Chemical, IL, USA). The stained protein bands were quantitatively analyzed using Image Lab software (version 5.1).

### 2.7 Statistical analysis

The data are presented as the means ± standard errors of the means (SEMs). One-way ANOVA followed by the unpaired Student's *t*-test or Dunnett's multiple comparison *post hoc* test was performed using SPSS 17.0. *p* < 0.05 was considered statistically significant.

## 3 Results

### 3.1 Content and composition of tea polyphenols

**Figure 1:**
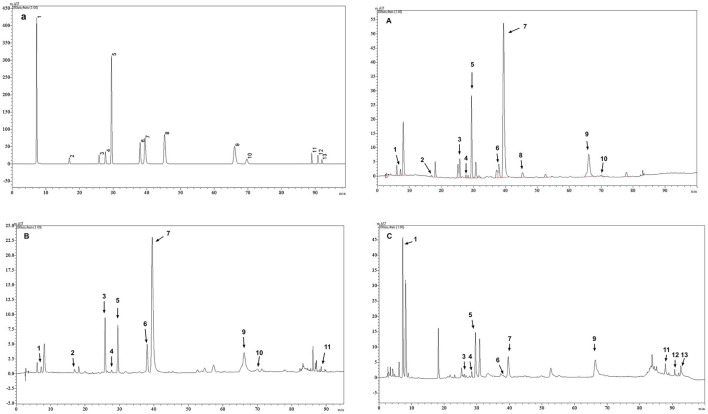
Representative HPLC chromatogram of (a) mixed standard solution (24); **(A)** tea phenolic extracts of Longjing green tea (24); **(B)** tea phenolic extracts of Tieguanyin oolong tea; **(C)** tea phenolic extracts of Dianhong black tea. The numbers of the peaks in this figure coincide with the compound numbers in Table 2.

The main phenolic substances detected in the polyphenolic extracts of Longjing green tea, Tieguanyin oolong tea and Dianhong black tea are shown in Table 2 and Figure 1. Among them, the subject of HPLC data of Longjing green tea has been published in other journals (24). The main components of the Longjing green tea polyphenolic extract included EGCG (288.83 μg/mg), which accounted for 28.8% of the total, and EGC (57.74 μg/mg), ECG (42.08 μg/mg), C, and CAF. The main components of the Tieguanyin oolong tea polyphenol extract included EGCG (129.21 μg/mg), which accounted for 12.9% of the total, EGC (71.49 μg/mg), EC (17.78 μg/mg), ECG (16.78 μg/mg), and C. The main components of the Dianhong black tea polyphenol extract included EGCG (34.70 μg/mg), ECG (30.42 μg/mg), GA (24.48 μg/mg), C, and TF. Among them, three polyphenol aggregates, TF, TF-3-G and TF-3′-G, were not detected in the polyphenol extract of Longjing; GCG, TF-3-G and TF-3′-G were not detected in the polyphenol extract of Tieguanyin; and GC, GCG and CG were not detected in the polyphenol extract of Dianhong. The validation of this HPLC analytical method did not include confirmation of the limit of detection (LOD) and limit of quantification (LOQ). Its core limitation lies in the inability to determine the sensitivity boundaries of the method.

**Table 2:** Qualitative and quantitative analysis of polyphenol monomers by HPLC.

**Serial number**	**Standard**	**Retention time (min)**	**Regression equation**	**Correlation coefficient**	**Longjing polyphenol concentration (μg/mg) (24)**	**TieGuanYin polyphenol concentration (μg/mg)**	**DianHong polyphenol concentration (μg/mg)**
1	GA	7.346	y = 22,543x + 408	1.000	1.37	0.66	24.48
2	GC	17.203	y = 2,094x – 425.1	0.999	4.95	4.70	ND
3	EGC	25.982	y = 1,927x – 2,148	0.999	57.74	71.49	6.75
4	C	27.929	y = 640.5x – 124.9	0.999	32.22	11.73	16.78
5	CAF	29.679	y = 20,816x + 3,051	0.999	21.69	5.95	11.32
6	EC	38.223	y = 5,898x – 645.1	1.000	21.14	17.40	1.89
7	EGCG	39.750	y = 5,958x – 11,652	0.994	288.83	129.21	34.70
8	GCG	45.569	y = 11,918x-54,003	0.990	9.47	ND	ND
9	ECG	66.456	y = 9,752x – 12,054	0.970	42.08	16.78	30.42
10	CG	69.977	y = 9,710x – 42,289	0.959	5.99	5.29	ND
11	TF	88.879	y = 4,074x + 1,046.6	0.999	ND	1.97	15.42
12	TF-3-G	90.708	y = 12,106x – 7,573	0.998	ND	ND	4.58
13	TF-3′-G	91.878	y = 16,418x + 11,688	0.999	ND	ND	11.84

ND = Not Detect indicates that the sample cannot be detected by the instrument under these experimental conditions.

### 3.2 Effects and mechanisms of action of the polyphenol extracts from three varieties of fermented teas on CYP450s in HepG2 cells

#### 3.2.1 Effects of tea polyphenol extracts on CYP3A4 expression

**Figure 2:**
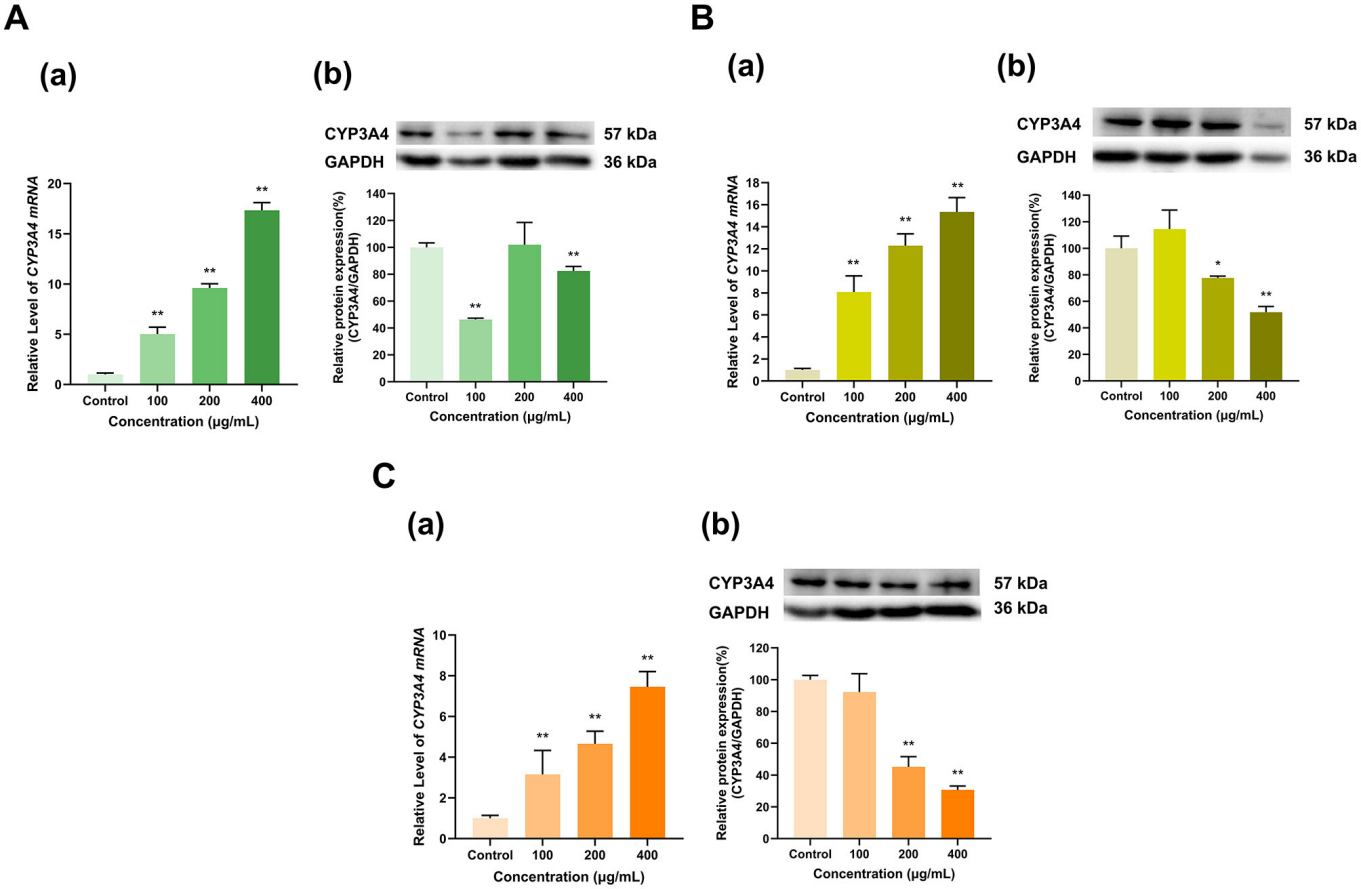
Effect of tea polyphenol extracts on CYP3A4 expression in HepG2 cells. **(A)** Effects of Longjing green tea polyphenolic extracts on the mRNA (a) and protein (b) levels of CYP3A4; **(B)** Effects of Tieguanyin oolong polyphenolic extracts on the mRNA (a) and protein (b) levels of CYP3A4; **(C)** Effects of Dianhong black tea polyphenolic extracts on the mRNA (a) and protein (b) levels of CYP3A4; The normalized expression data are presented as the mean ± SD (*n* = 3). *p < 0.05 vs. control group, **p < 0.01 vs. control group.

Our results revealed that the polyphenol extracts from these three teas with varying levels of fermentation significantly upregulated CYP3A4 mRNA but dramatically inhibited its overall protein expression compared with that in the control group (Figure 2). Among them, the upregulation of CYP3A4 mRNA by the polyphenol extracts of Longjing green tea, Tieguanyin oolong tea and Dianhong black tea gradually increased with increasing extract concentration (100, 200, and 400 μg/ml). Specifically, CYP3A4 mRNA expression was 5.02, 9.62, and 17.34; 8.08, 12.29, and 15.35; and 3.16, 4.66, and 7.46 times greater in the three tea extract groups at the three concentrations than that in the control group, respectively (*p* < 0.01 for all). In contrast, Longjing green tea polyphenol extract at doses of 100 μg/ml and 400 μg/ml decreased CYP3A4 protein expression to ~46% and 82%, respectively, of that in the control group (*p* < 0.01, *p* < 0.01), although 200 μg/ml had no significant effect on protein expression. Similarly, at 100 μg/ml, the Tieguanyin oolong tea and Dianhong black tea polyphenol extracts had no significant effect on CYP3A4 protein expression. At concentrations of 200 μg/ml and 400 μg/ml, the polyphenol extracts of Tieguanyin oolong tea and Dianhong black tea significantly reduced CYP3A4 protein expression to 78% and 52%; and 45% and 31% of that in the control group (*p* < 0.05, *p* < 0.01, *p* < 0.01, *p* < 0.01), respectively. Taken together, these findings indicate that tea polyphenols increase the mRNA expression of the CYP3A4 enzyme but inhibit its protein expression.

#### 3.2.2 Effects of tea polyphenol extracts on CYP2E1 expression

**Figure 3:**
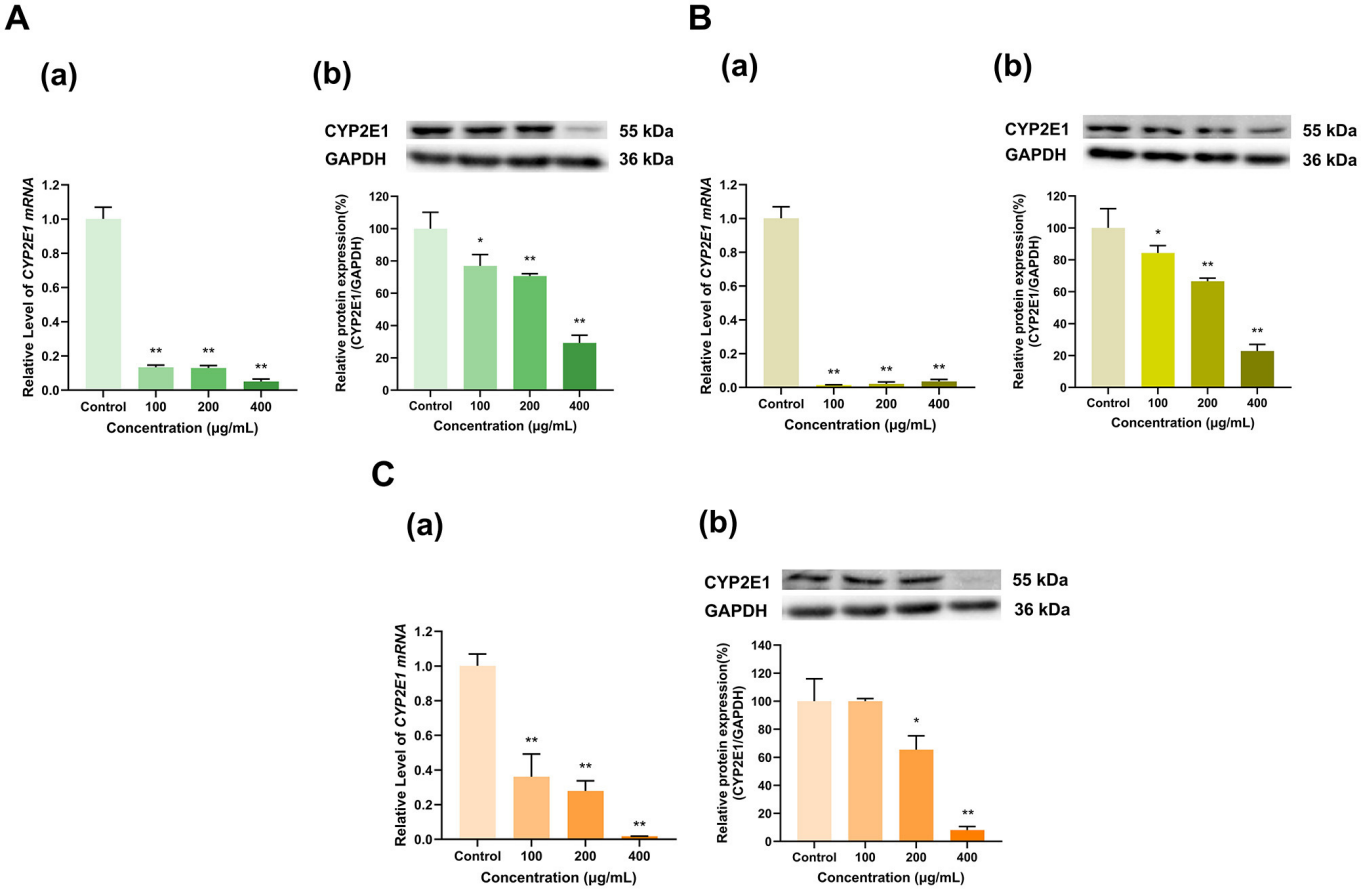
Effect of tea polyphenol extracts on CYP2E1 expression in HepG2 cells. **(A)** Effects of Longjing green tea polyphenolic extracts on the mRNA (a) and protein (b) levels of CYP2E1; **(B)** Effects of Tieguanyin oolong polyphenolic extracts on the mRNA (a) and protein (b) levels of CYP2E1; **(C)** Effects of Dianhong black tea polyphenolic extracts on the mRNA (a) and protein (b) levels of CYP2E1; The normalized expression data are presented as the mean ± SD (*n* = 3). *p < 0.05 vs. control group, **p < 0.01 vs. control group.

As shown in Figure 3, the three tea polyphenol extracts with different degrees of fermentation significantly inhibited CYP2E1 expression. Specifically, different doses (100, 200, and 400 μg/ml) of the Longjing green tea polyphenol extract decreased CYP2E1 mRNA expression to 13%, 13%, and 5%, respectively, and CYP2E1 protein expression to 77%, 71%, and 29%, respectively, of that in the control group (*p* < 0.01, *p* < 0.01, *p* < 0.01, *p* < 0.05, *p* < 0.01, *p* < 0.01). Similarly, the all doses (100, 200, and 400 μg/ml) of Tieguanyin oolong tea polyphenol extract decreased CYP2E1 mRNA expression to 1%, 2%, and 4%, respectively, and CYP2E1 protein expression to 84%, 67%, and 23%, respectively, of that in the control group (*p* < 0.01, *p* < 0.01, *p* < 0.01, *p* < 0.05, *p* < 0.01, *p* < 0.01), exhibiting dose-dependent effects (Figure 3B). In the 100 μg/ml Dianhong black tea polyphenol extract-treated group, CYP2E1 mRNA expression was 36% that in the control group but CYP2E1 protein expression did not change; furthermore, CYP2E1 mRNA and protein expression levels decreased to 28% and 65% of those in the control group at a concentration of 200 μg/ml and 2% and 8% of those in the control group at a concentration of 400 μg/ml (*p* < 0.01, *p* < 0.05, *p* < 0.01, *p* < 0.01), respectively, also exhibiting dose-dependent effects (Figure 3C). Overall, CYP2E1 expression was inhibited by the tea extracts.

#### 3.2.3 Effects of tea polyphenol extracts on CYP2C9 expression

**Figure 4:**
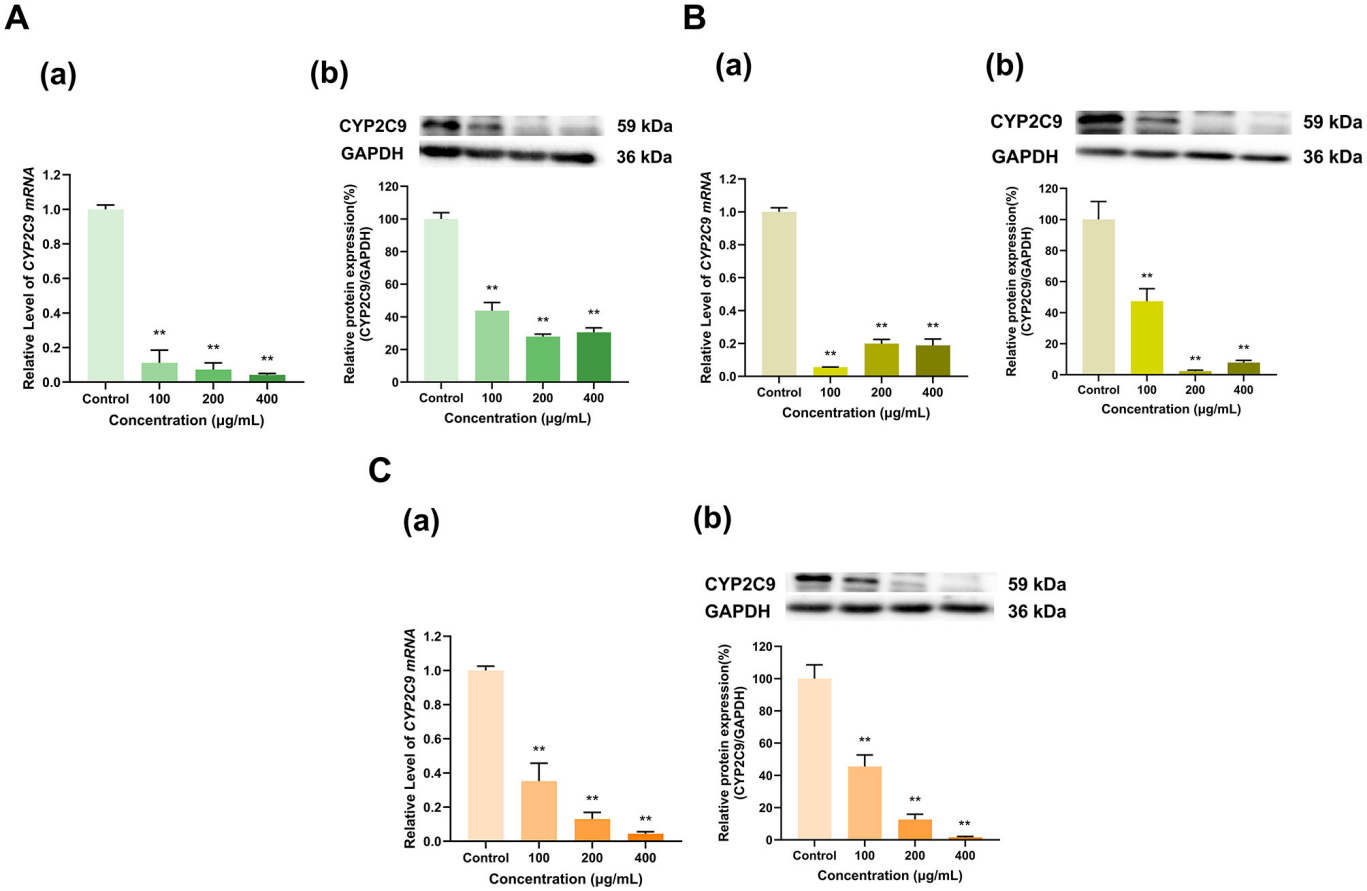
Effect of tea polyphenol extracts on CYP2C9 expression in HepG2 cells. **(A)** Effects of Longjing green tea polyphenolic extracts on the mRNA (a) and protein (b) levels of CYP2C9; **(B)** Effects of Tieguanyin oolong polyphenolic extracts on the mRNA (a) and protein (b) levels of CYP2C9; **(C)** Effects of Dianhong black tea polyphenolic extracts on the mRNA (a) and protein (b) levels of CYP2C9; The normalized expression data are presented as the mean ± SD (*n* = 3). **p < 0.01 vs. control group.

Compared with the control group, the polyphenol extracts from three types of tea with different degrees of fermentation significantly inhibited both the mRNA and protein expression of CYP2C9 in HepG2 cells (Figure 4). The Longjing green tea polyphenol extracts (100, 200, and 400 μg/ml) downregulated both CYP2C9 mRNA to 11%, 7%, and 4%, respectively, of that in the control group (*p* < 0.01 for all) and CYP2C9 protein to 44%, 28%, and 31%, respectively, of that in the control group (*p* < 0.01 for all) (Figure 4A). Similarly, the Tieguanyin oolong tea polyphenol extracts (100, 200, and 400 μg/ml) decreased CYP2C9 mRNA expression to 6%, 20%, and 19%, respectively, of that in the control group (*p* < 0.01 for all) and CYP2C9 protein expression to 47%, 2%, and 8%, respectively, of that in the control group (*p* < 0.01 for all) (Figure 4B). In particular, the inhibitory effect of the Dianhong black tea polyphenol extracts on CYP2C9 gradually increased with increasing dose. Specifically, CYP2C9 mRNA expression was reduced to 35%, 13%, and 4% of that in the control group in the 100, 200, and 400 μg/ml Dianhong black tea polyphenol extract dose groups (*p* < 0.01 for all), respectively, and protein expression was reduced to 45%, 13%, and 2% of that in the control group, respectively (*p* < 0.01 for all) (Figure 4C). Overall, CYP2C9 expression was inhibited by the tea extracts.

#### 3.2.4 Effects of tea polyphenol extracts on CYP1A2 expression

**Figure 5:**
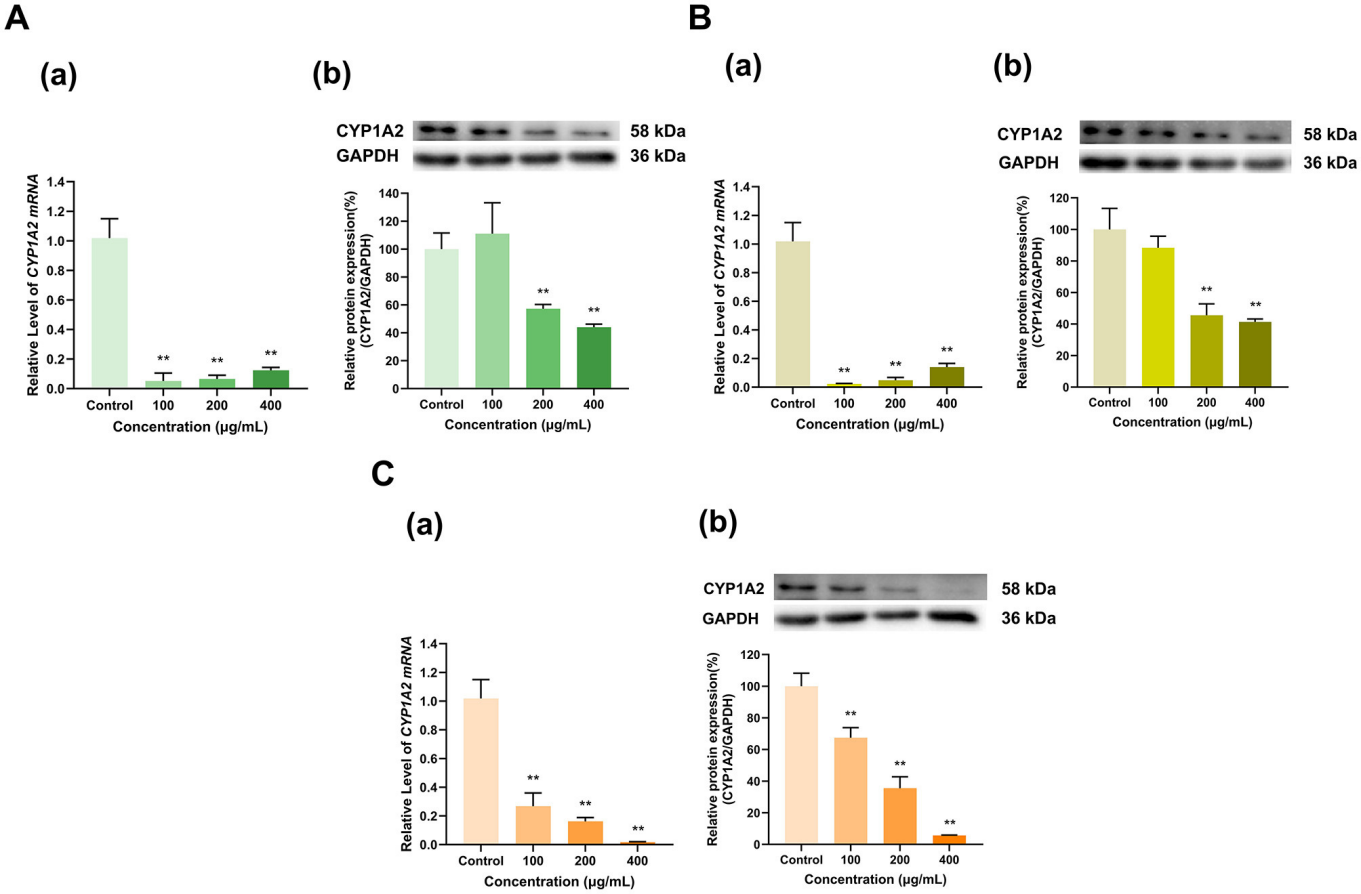
Effect of tea polyphenol extracts on CYP1A2 expression in HepG2 cells. **(A)** Effects of Longjing green tea polyphenolic extracts on the mRNA (a) and protein (b) levels of CYP1A2; **(B)** Effects of Tieguanyin oolong polyphenolic extracts on the mRNA (a) and protein (b) levels of CYP1A2; **(C)** Effects of Dianhong black tea polyphenolic extracts on the mRNA (a) and protein (b) levels of CYP1A2; The normalized expression data are presented as the mean ± SD (*n* = 3). **p < 0.01 vs. control group.

As shown in Figure 5, the three tea polyphenol extracts with different degrees of fermentation significantly inhibited CYP1A2 expression overall. Specifically, the 100 μg/ml doses of Longjing green tea and Tieguanyin oolong tea polyphenol extracts inhibited CYP1A2 mRNA expression to 5% and 2% of that in the control group, respectively (*p* < 0.01 for all), but had no significant effect on CYP1A2 protein expression. At doses of 200 and 400 μg/ml, the Longjing green tea polyphenol extract reduced CYP1A2 mRNA expression to 6% and 12% and CYP1A2 protein expression to 57% and 44%, respectively, of that in the control group (*p* < 0.01 for all) (Figure 5A). Moreover, the Tieguanyin oolong tea polyphenol extract decreased CYP1A2 mRNA expression to 5% and 14% and CYP1A2 protein expression to 46% and 41%, respectively, of that in the control group (*p* < 0.01 for all) (Figure 5B). In addition, the Dianhong black tea polyphenol extract inhibited CYP1A2 expression in a dose-dependent manner, with the 100, 200, and 400 μg/ml doses decreasing CYP1A2 mRNA expression to 27%, 16%, and 2% of that in the control group, respectively, and decreasing CY1A2 protein expression to 67%, 36%, and 6% of that in the control group, respectively (*p* < 0.01 for all) (Figure 5C). Overall, CYP1A2 expression was inhibited by the tea extracts.

### 3.3 Effects and mechanisms of action of EGCG, EGC, and ECG on CYP450s in HepG2 cells

#### 3.3.1 Effects of EGCG, EGC and ECG on CYP3A4 expression

**Figure 6:**
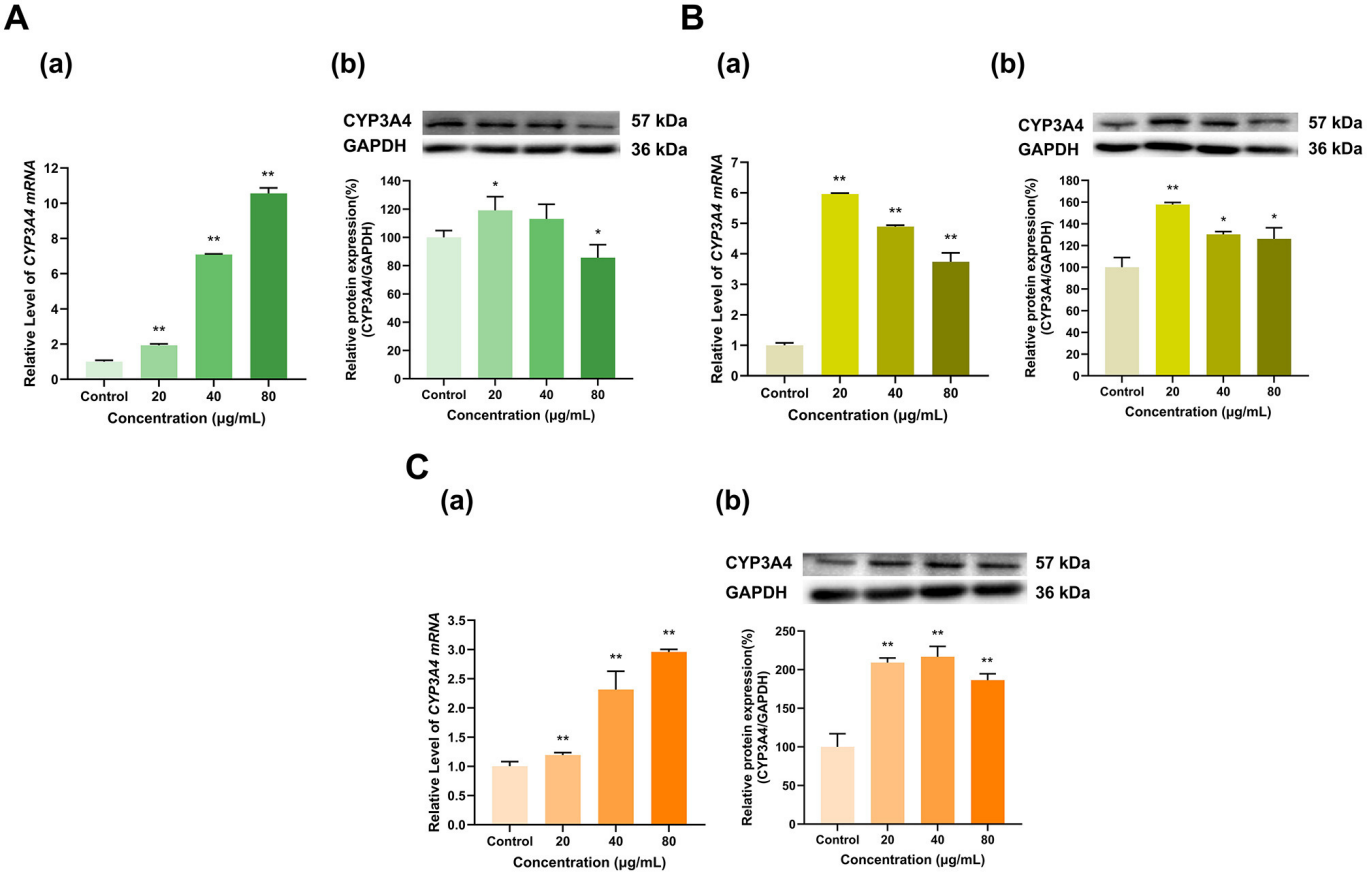
Effect of Catechins on CYP3A4 expression in HepG2 cells. **(A)** Effects of EGCG on the mRNA (a) and protein (b) levels of CYP3A4; **(B)** Effects of EGC on the mRNA (a) and protein (b) levels of CYP3A4; **(C)** Effects of ECG on the mRNA (a) and protein (b) levels of CYP3A4; The normalized expression data are presented as the mean ± SD (*n* = 3). *p < 0.05 vs. control group, **p < 0.01 vs. control group.

Overall, EGCG, EGC and ECG significantly induced CYP3A4 expression (Figure 6). CYP3A4 mRNA expression was induced by EGCG in a dose-dependent manner; at doses of 20, 40, and 80 μg/ml, CYP3A4 mRNA expression was 1.9, 7.1, and 10.6 times greater than that in the control group, respectively (*p* < 0.01 for all) (Figure 6A). The effect of EGCG on the CYP3A4 protein expression level transitioned from induction to inhibition with increasing dose; with respect to that in the control group, CYP3A4 protein expression in the 20 μg/ml group increased to 119% (*p* < 0.05), that in the 40 μg/ml dose group did not significantly change, and that in the 80 μg/ml dose group decreased to 86% that of the control group (*p* < 0.05). EGC at different doses (20, 40, and 80 μg/ml) increased CYP3A4 mRNA expression to 6, 4.9, and 3.7 times that in the control group (*p* < 0.01 for all) and increased CYP3A4 protein expression to 158%, 130%, and 126% of that in the control group (*p* < 0.01, *p* < 0.05, *p* < 0.05), respectively (Figure 6B). Similarly, ECG at different doses (20, 40, and 80 μg/ml) increased CYP3A4 mRNA expression to 1.2, 2.3, and 3 times that in the control group (*p* < 0.01 for all) and increased CYP3A4 protein expression to 209%, 217%, and 186% of that in the control group, respectively (*p* < 0.01 for all) (Figure 6C). Overall, CYP3A4 expression was induced by EGCG, EGC and ECG.

#### 3.3.2 Effects of EGCG, EGC and ECG on CYP2E1 expression

**Figure 7:**
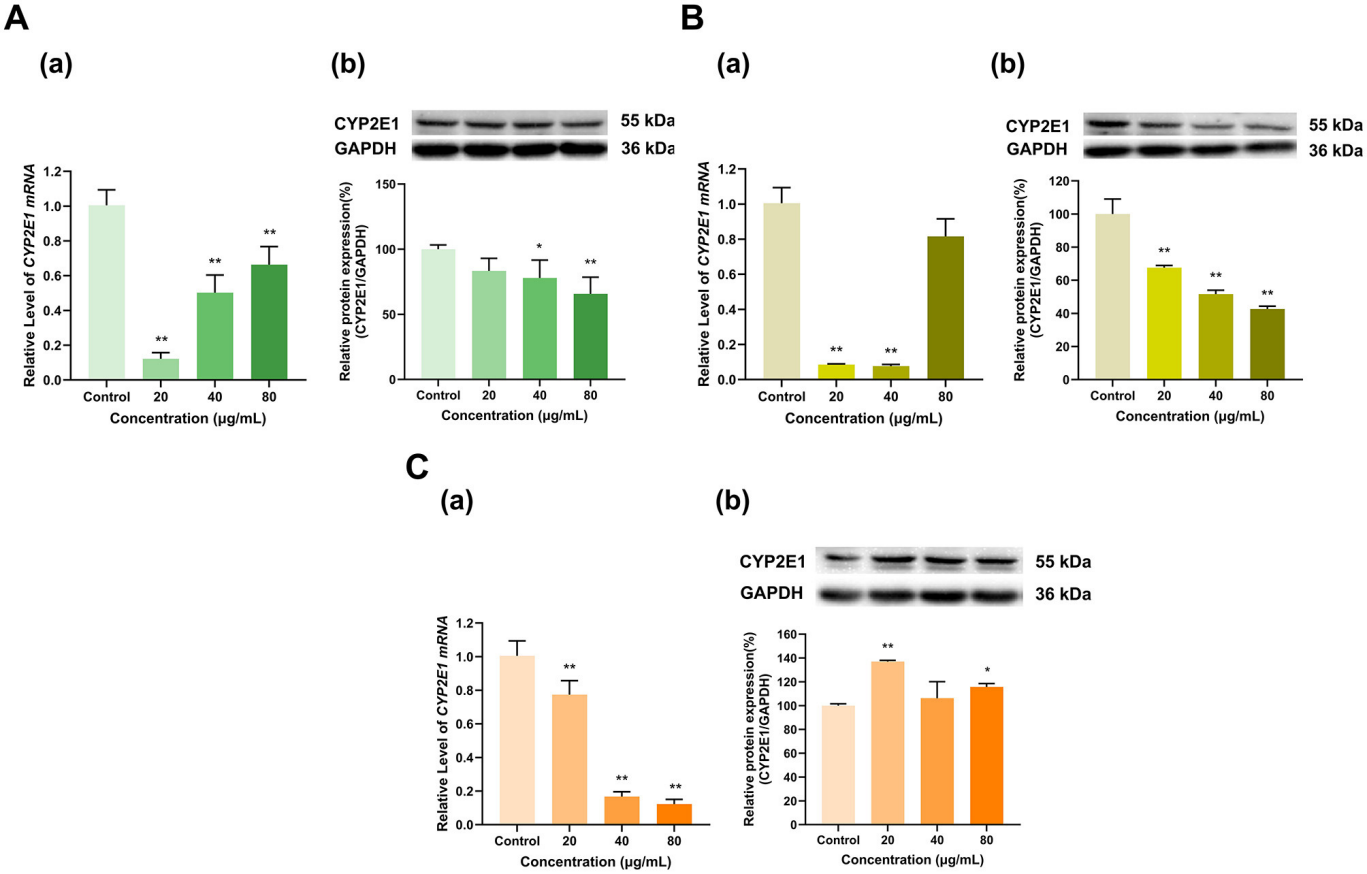
Effect of Catechins on CYP2E1 expression in HepG2 cells. **(A)** Effects of EGCG on the mRNA (a) and protein (b) levels of CYP2E1; **(B)** Effects of EGC on the mRNA (a) and protein (b) levels of CYP2E1; **(C)** Effects of ECG on the mRNA (a) and protein (b) levels of CYP2E1; The normalized expression data are presented as the mean ± SD (*n* = 3). *p < 0.05 vs. control group, **p < 0.01 vs. control group.

As shown in Figure 7, overall, EGCG, EGC, and ECG inhibited the expression of CYP2E1. EGCG at doses of 20, 40, and 80 μg/ml downregulated CYP2E1 mRNA to 12%, 50%, and 66%, respectively, of that in the control group (*p* < 0.01 for all). EGCG at 20 μg/ml had no significant effect on CYP2E1 protein expression, while EGCG at 40 and 80 μg/ml inhibited CYP2E1 protein expression to 78% and 66% of that in the control group (*p* < 0.05, *p* < 0.01), respectively (Figure 7A). At 20 and 40 μg/ml, EGC downregulated CYP2E1 mRNA to 8% and 7% of that in the control group, respectively, and at 80 μg/ml, EGC had no significant effect on CYP2E1 mRNA expression (Figure 7B). CYP2E1 protein expression decreased to 68%, 52%, and 43% of that in the control group at EGC doses of 20, 40, and 80 μg/ml, respectively (*p* < 0.01 for all). ECG at doses of 20, 40, and 80 μg/ml downregulated CYP2E1 mRNA to 77%, 17% and 12% of that in the control group, respectively (*p* < 0.01 for all). However, at doses of 20 and 80 μg/ml, ECG increased CYP2E1 protein expression to 137% and 116% of that in the control group (*p* < 0.01, *p* < 0.05), respectively, while the 40 μg/ml dose had no significant effect on CYP2E1 protein expression (Figure 7C). Overall, the effects of EGCG, EGC, and ECG on CYP2E1 expression were inhibitory.

#### 3.3.3 Effects of EGCG, EGC and ECG on CYP2C9 expression

**Figure 8:**
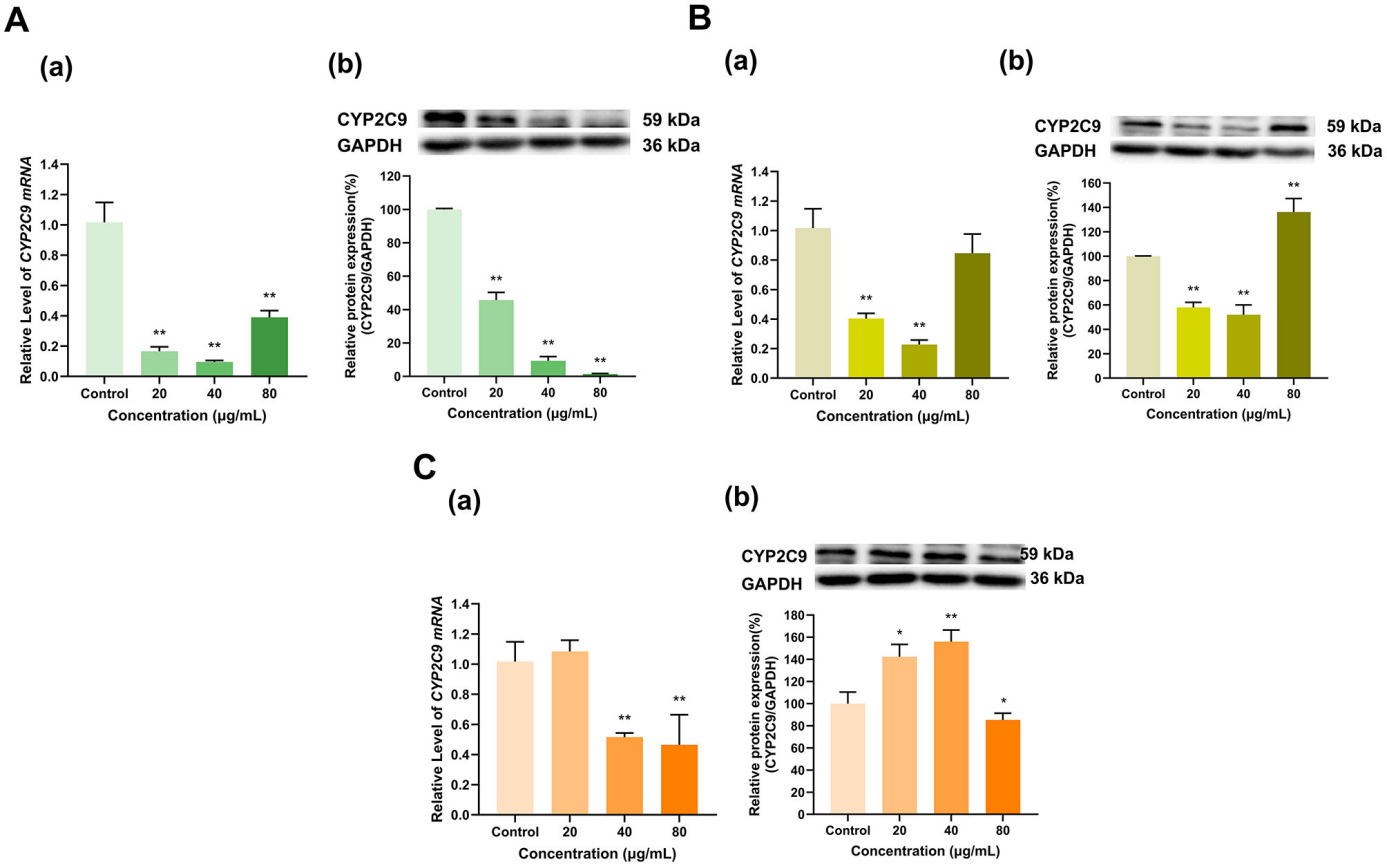
Effect of Catechins on CYP2C9 expression in HepG2 cells. **(A)** Effects of EGCG on the mRNA (a) and protein (b) levels of CYP2C9; **(B)** Effects of EGC on the mRNA (a) and protein (b) levels of CYP2C9; **(C)** Effects of ECG on the mRNA (a) and protein (b) levels of CYP2C9; The normalized expression data are presented as the mean ± SD (*n* = 3). *p < 0.05 vs. control group, **p < 0.01 vs. control group.

As shown in Figure 8, overall, EGCG, EGC and ECG significantly inhibited CYP2C9 expression. EGCG at doses of 20, 40, and 80 μg/ml lowered the expression of CYP2C9 mRNA to 17%, 10%, and 39% of that in the control group, respectively (*p* < 0.01 for all), and decreased its protein expression to 46%, 9%, and 1% of that in the control group, respectively (*p* < 0.01 for all) (Figure 8A). The 20 and 40 μg/ml doses of EGC downregulated CYP2C9 mRNA to 40% and 23% of that in the control group, respectively (*p* < 0.01 for all) and decreased its protein expression to 58% and 52% of that in the control group, respectively (*p* < 0.01 for all). Moreover, whereas the 80 μg/ml dose of EGC did not significantly affect CYP2C9 mRNA expression but increased its protein expression to 136% of that in the control group (*p* < 0.01) (Figure 8B). ECG at 20 μg/ml had no significant effect on CYP2C9 mRNA expression, but at doses of 40 and 80 μg/ml, the CYP2C9 expression level decreased to 52% and 46% of that in the control group, respectively (*p* < 0.01 for all). ECG at 20 and 40 μg/ml increased CYP2C9 protein expression to 142% and 156% of that in the control group (*p* < 0.05, *p* < 0.01), respectively, and ECG at 80 μg/ml decreased CYP2C9 protein expression to 85% of that in the control group (*p* < 0.05). Overall, the inhibitory effects of EGCG, EGC and ECG on CYP2C9 expression were dominant (Figure 8C).

#### 3.3.4 Effects of EGCG, EGC and ECG on CYP1A2 expression

**Figure 9:**
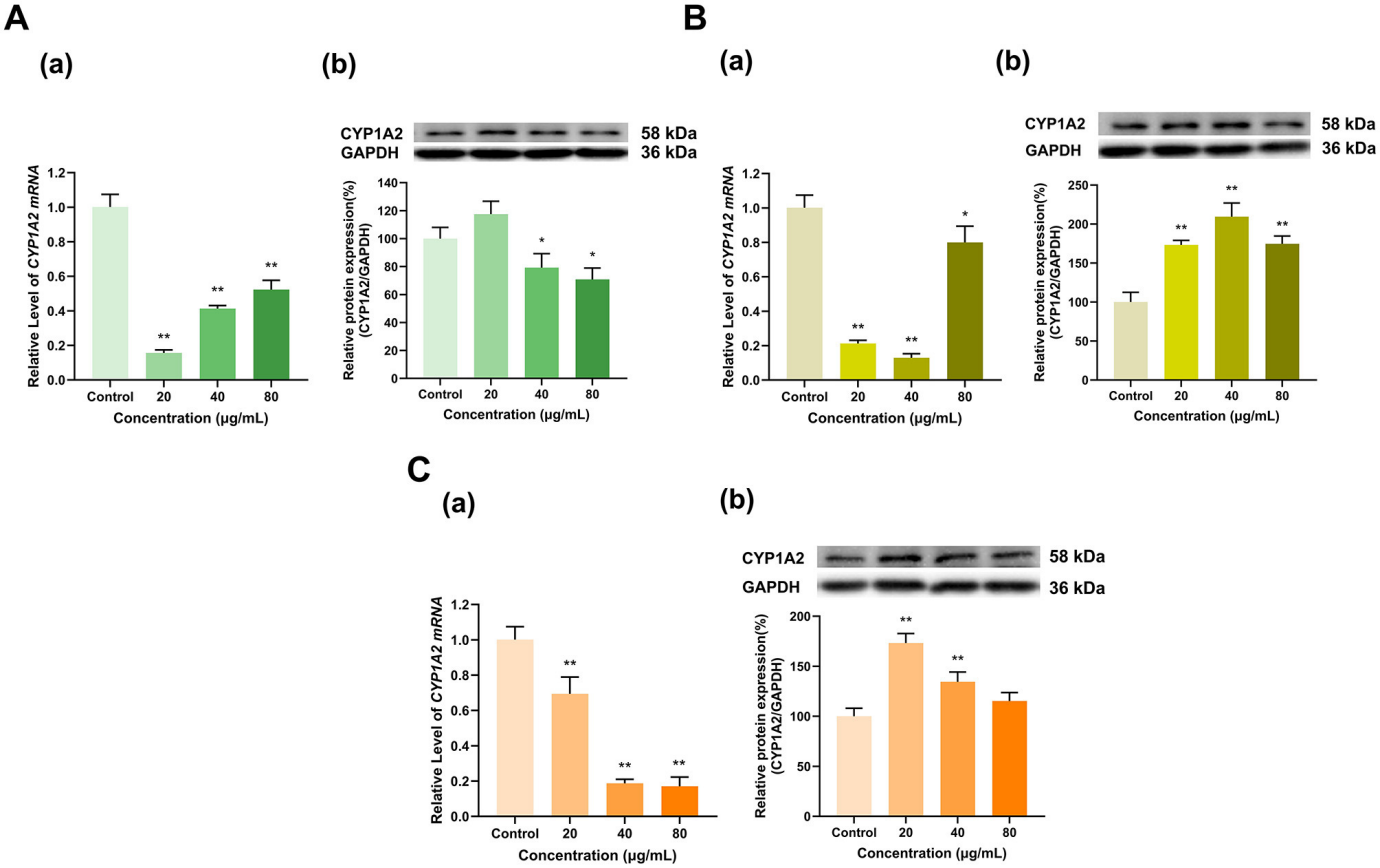
Effect of Catechins on CYP1A2 expression in HepG2 cells. **(A)** Effects of EGCG on the mRNA (a) and protein (b) levels of CYP1A2; **(B)** Effects of EGC on the mRNA (a) and protein (b) levels of CYP1A2; **(C)** Effects of ECG on the mRNA (a) and protein (b) levels of CYP1A2; The normalized expression data are presented as the mean ± SD (*n* = 3). *p < 0.05 vs. control group, **p < 0.01 vs. control group.

EGCG had an overall inhibitory effect on CYP1A2 expression (Figure 9A). Specifically, CYP1A2 mRNA expression was decreased to 16%, 41% and 52% of that in the control group at EGCG doses of 20, 40 and 80 μg/ml, respectively (p < 0.01 for all), and EGCG at 40 and 80 μg/ml decreased CYP1A2 protein expression to 79% and 71% of that in the control group, respectively (p < 0.05 for all), whereas no significant changes were observed at 20 μg/ml. EGC and ECG inhibited CYP1A2 mRNA expression and induced its protein expression. EGC at 20, 40, and 80 μg/ml decreased CYP1A2 mRNA expression to 21%, 13%, and 80% of that in the control group (*p* < 0.01, p < 0.01, *p* < 0.05), respectively, but increased its protein expression to 173%, 209%, and 175% of that in the control group, respectively (*p* < 0.01 for all) (Figure 9B). Similarly, ECG at 20, 40, and 80 μg/ml lowered CYP1A2 mRNA expression to 69%, 19%, and 17% of that in the control group, respectively (*p* < 0.01 for all), and at 20 and 40 μg/ml, ECG increased CYP1A2 protein expression to 173% and 134% that in the control group, respectively (*p* < 0.01 for all), while ECG at 80 μg/ml had no significant effect on CYP1A2 protein expression (Figure 9C).

### 3.4 Effects and mechanisms of action of TF, TF-3-G, and TF-3′-G on CYP450s in HepG2 cells

#### 3.4.1 Effects of TF, TF-3-G, and TF-3′-G on CYP3A4 expression

**Figure 10:**
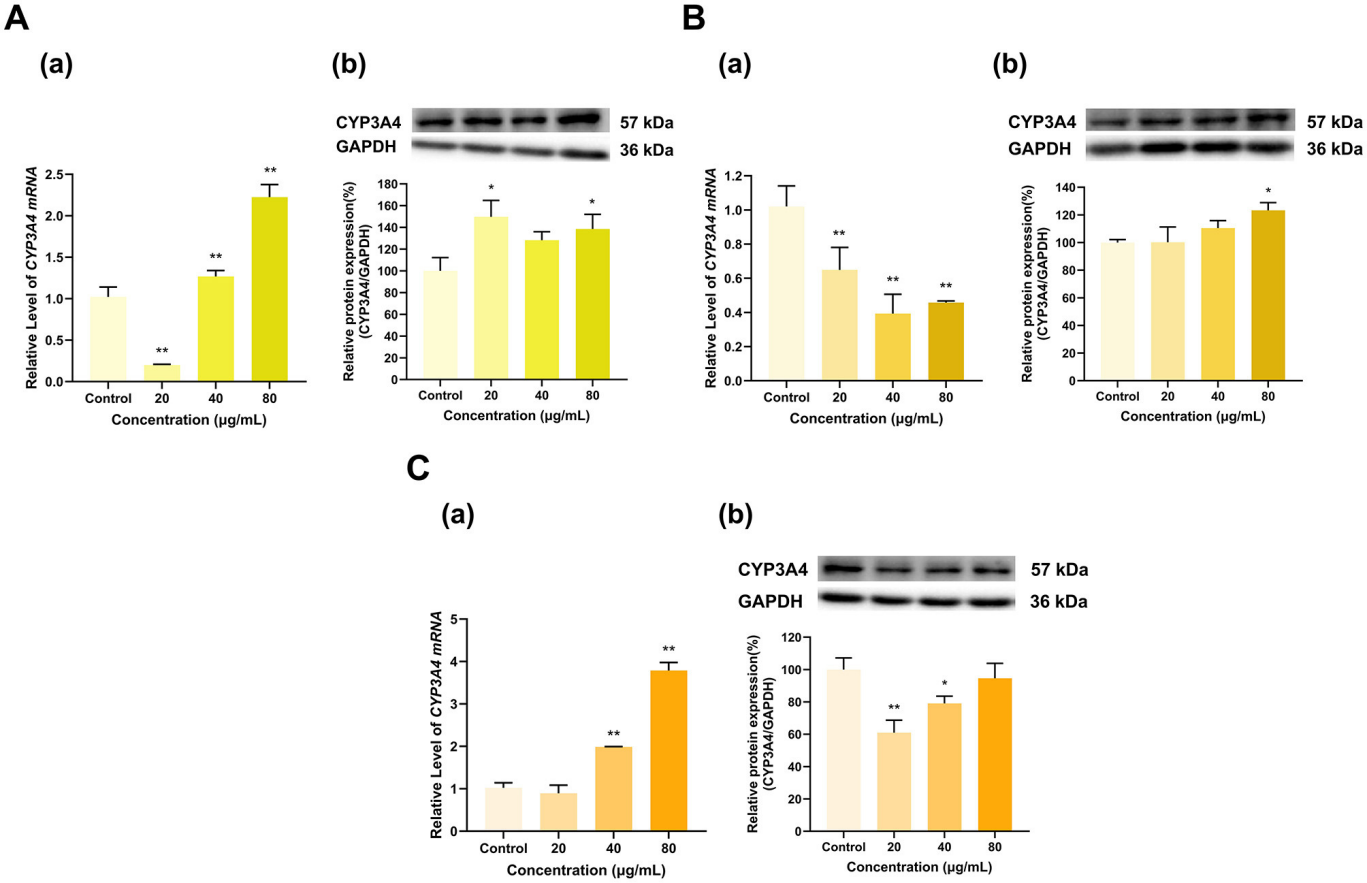
Effect of Theaflavins on CYP3A4 expression in HepG2 cells. **(A)** Effects of TF on the mRNA (a) and protein (b) levels of CYP3A4; **(B)** Effects of TF-3-G on the mRNA (a) and protein (b) levels of CYP3A4; **(C)** Effects of TF-3′-G on the mRNA (a) and protein (b) levels of CYP3A4; The normalized expression data are presented as the mean ± SD (*n* = 3). *p < 0.05 vs. control group, **p < 0.01 vs. control group.

TF, TF-3-G and TF-3′-G all significantly affected CYP3A4 expression (Figure 10). Overall, TF induced the expression of CYP3A4 (Figure 10A). At 20 μg/ml, TF decreased CYP3A4 mRNA expression to 20% of that in the control group and increased its protein expression to 150% of that in the control group (*p* < 0.01, *p* < 0.05); at 40 μg/ml, TF increased the expression of CYP3A4 mRNA to 1.3 times that in the control group but did not have a significant effect on its protein expression (*p* < 0.01); and at 80 μg/ml, TF increased the expression of CYP3A4 mRNA to 2.2 times that in the control group and increased its protein expression to 138% (*p* < 0.01, *p* < 0.05). TF-3-G at 20, 40, and 80 μg/ml decreased the expression of CYP3A4 mRNA to 65%, 39%, and 46% of that in the control group, respectively (*p* < 0.01 for all). At 80 μg/ml, TF-3-G increased CYP3A4 protein expression to 123% of that in the control group (*p* < 0.05), whereas the 20 and 40 μg/ml doses of TF-3-G groups had no significant effect on CYP3A4 protein expression (Figure 10B). At 40 and 80 μg/ml, TF-3′-G increased the expression of CYP3A4 mRNA to 2 and 3.8 times that in the control group, respectively (*p* < 0.01 for all), whereas 20 μg/ml TF-3′-G had no significant effect on CYP3A4 mRNA expression. At 20 and 40 μg/ml, TF-3′-G decreased the expression of CYP3A4 protein to 61% and 79% of that in the control group (*p* < 0.01, *p* < 0.05), respectively, whereas 80 μg/ml TF-3′-G had no significant effect on the protein expression of CYP3A4 (Figure 10C).

#### 3.4.2 Effects of TF, TF-3-G, and TF-3′-G on CYP2E1 expression

**Figure 11:**
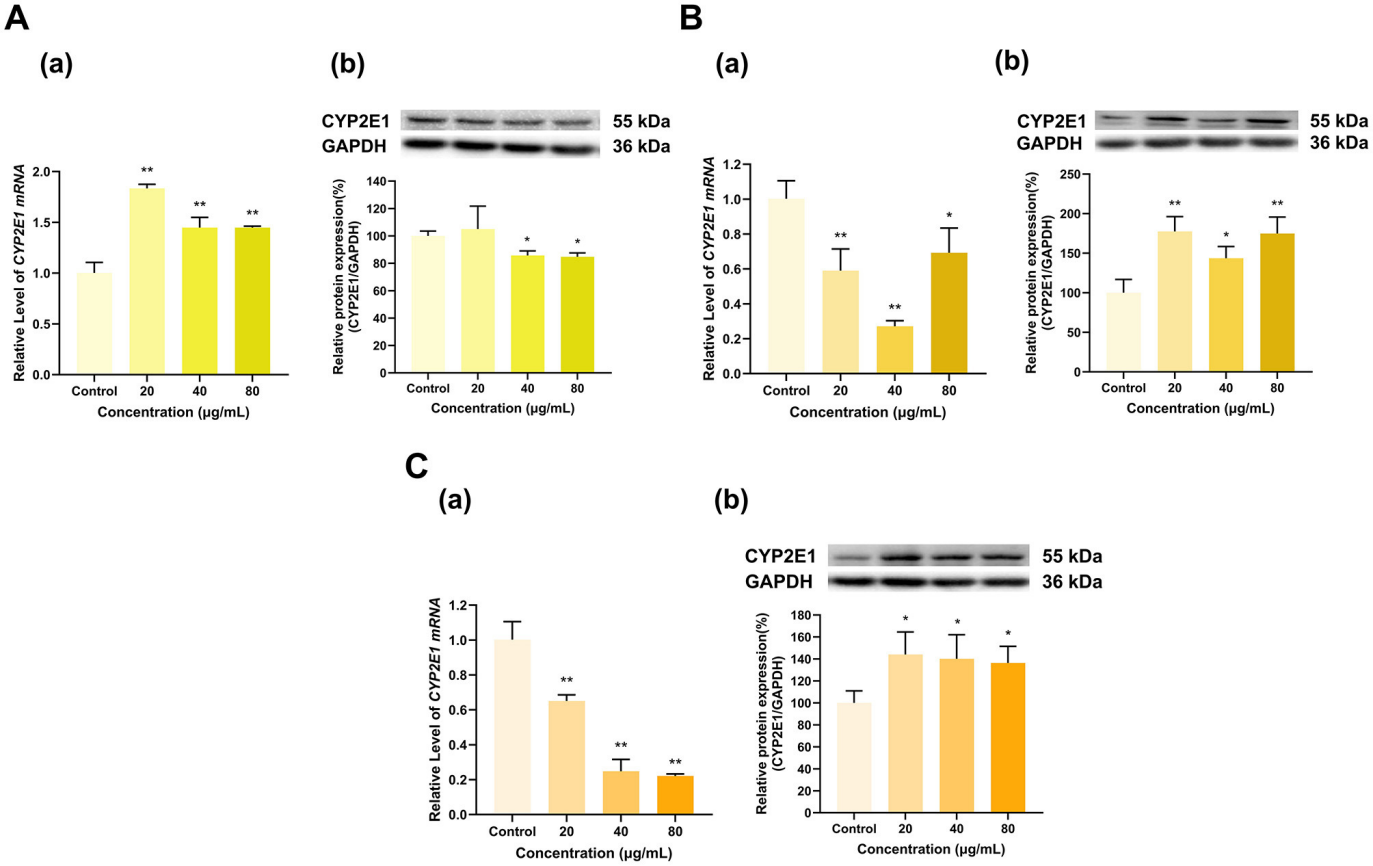
Effect of Theaflavins on CYP2E1 expression in HepG2 cells. **(A)** Effects of TF on the mRNA (a) and protein (b) levels of CYP2E1; **(B)** Effects of TF-3-G on the mRNA (a) and protein (b) levels of CYP2E1; **(C)** Effects of TF-3′-G on the mRNA (a) and protein (b) levels of CYP2E1; The normalized expression data are presented as the mean ± SD (*n* = 3). *p < 0.05 vs. control group, **p < 0.01 vs. control group.

TF, TF-3-G and TF-3′-G significantly affected CYP2E1 expression (Figure 11). TF at 20, 40, and 80 μg/ml increased CYP2E1 mRNA expression to 1.8, 1.4, and 1.4 times that in the control group, respectively (*p* < 0.01 for all), and at 40 and 80 μg/ml, TF decreased CYP2E1 protein expression to 86% and 85% of that in the control group, respectively (*p* < 0.05 for all), while 20 μg/ml TF had no significant effect on CYP2E1 protein expression (Figure 11A). TF-3-G and TF-3′-G inhibited CYP2E1 mRNA expression but induced CYP2E1 protein expression. Specifically, at 20, 40, and 80 μg/ml, TF-3-G decreased CYP2E1 mRNA expression to 59%, 27%, and 69% of that in the control group (*p* < 0.01, *p* < 0.01, *p* < 0.05), respectively, and increased its protein expression to 178%, 144%, and 175% (*p* < 0.01, *p* < 0.05, *p* < 0.01), respectively (Figure 11B). Similarly, at 20, 40, and 80 μg/ml, TF-3′-G decreased the expression of CYP2E1 mRNA to 65%, 25%, and 22% of that in the control group, respectively (*p* < 0.01 for all) and increased its protein expression to 144%, 140%, and 136%, respectively (*p* < 0.05 for all) (Figure 11C).

#### 3.4.3 Effects of TF, TF-3-G, and TF-3′-G on CYP2C9 expression

**Figure 12:**
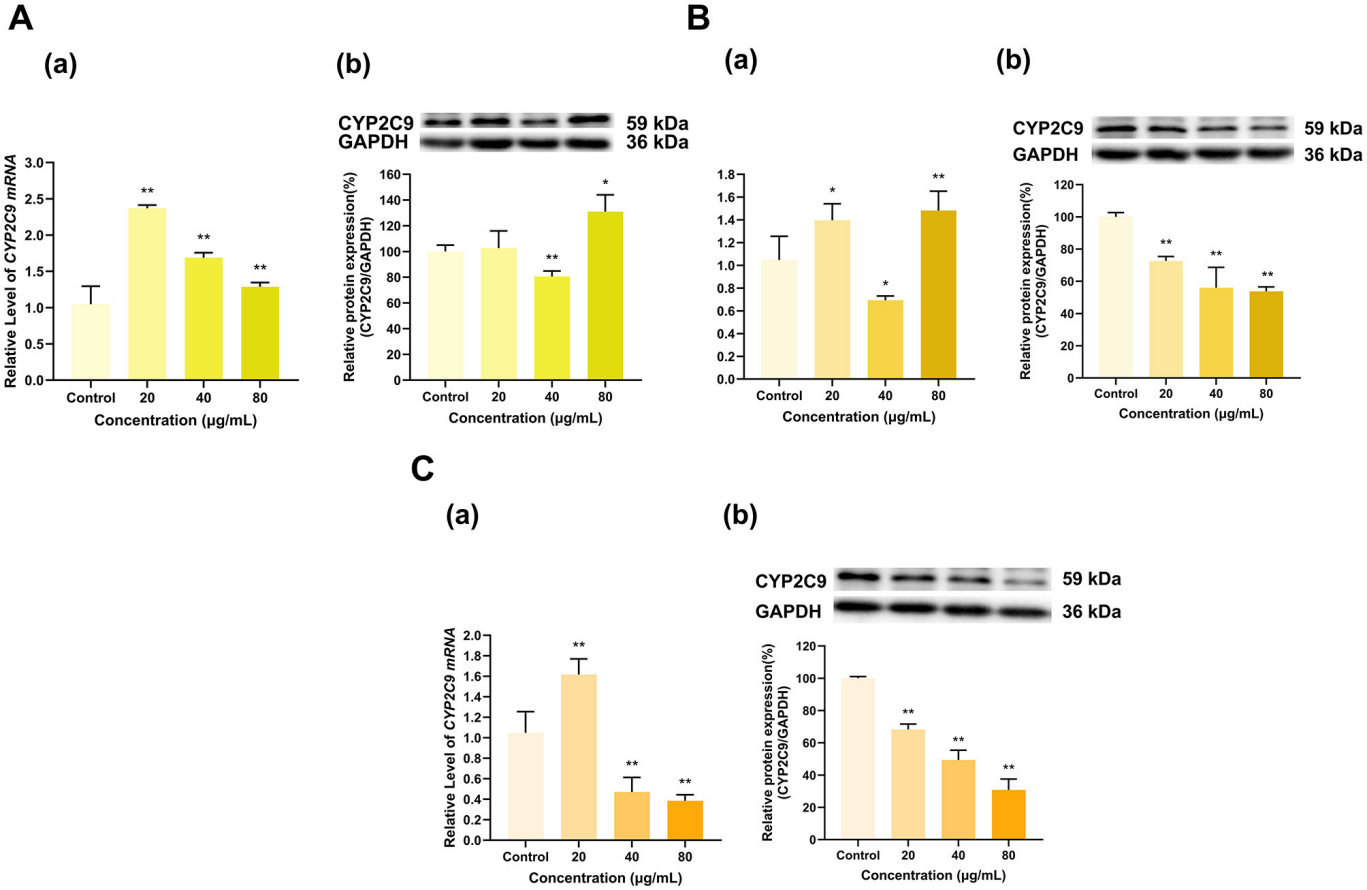
Effect of Theaflavins on CYP2C9 expression in HepG2 cells. **(A)** Effects of TF on the mRNA (a) and protein (b) levels of CYP2C9; **(B)** Effects of TF-3-G on the mRNA (a) and protein (b) levels of CYP2C9; **(C)** Effects of TF-3′-G on the mRNA (a) and protein (b) levels of CYP2C9; The normalized expression data are presented as the mean ± SD (*n* = 3). *p < 0.05 vs. control group, **p < 0.01 vs. control group.

As shown in Figure 12, TF, TF-3-G and TF-3′-G significantly affected CYP2C9 expression, notably inhibiting its protein expression (Figure 12). TF at 20, 40, and 80 μg/ml increased CYP2C9 mRNA expression to 2.4, 1.7 and 1.3 times that in the control group, respectively (*p* < 0.01 for all). At 40 μg/ml, TF decreased CYP2C9 protein expression to 81% of that in the control group (*p* < 0.01); at 80 μg/ml, TF increased CYP2C9 protein expression to 131% of the control group (*p* < 0.05); whereas at 20 μg/ml, TF had no significant effect on CYP2C9 protein expression (Figure 12A). TF-3-G at 20 and 80 μg/ml increased CYP2C9 mRNA expression to 1.4 and 1.5 times that in the control group (*p* < 0.05, *p* < 0.01), respectively, and at 40 μg/ml, TF-3-G decreased CYP2C9 mRNA expression to 69% of that in the control group (*p* < 0.05). At 20, 40, and 80 μg/ml, TF-3-G decreased the CYP2C9 protein expression level to 73%, 56%, and 54% of that in the control group, respectively (*p* < 0.01 for all) (Figure 12B). Similarly, TF-3′-G at 20 μg/ml increased CYP2C9 mRNA expression to 1.6 times that in the control group (*p* < 0.01), and at 40 and 80 μg/ml, TF-3′-G decreased CYP2C9 mRNA expression to 47% and 38% of that in the control group, respectively (*p* < 0.01 for all). At 20, 40, and 80 μg/ml, TF-3′-G inhibited the protein expression of CYP2C9 to 68%, 49%, and 31% of that in the control group, respectively (*p* < 0.01 for all) (Figure 12C).

#### 3.4.4 Effects of TF, TF-3-G, and TF-3′-G on CYP1A2 expression

**Figure 13:**
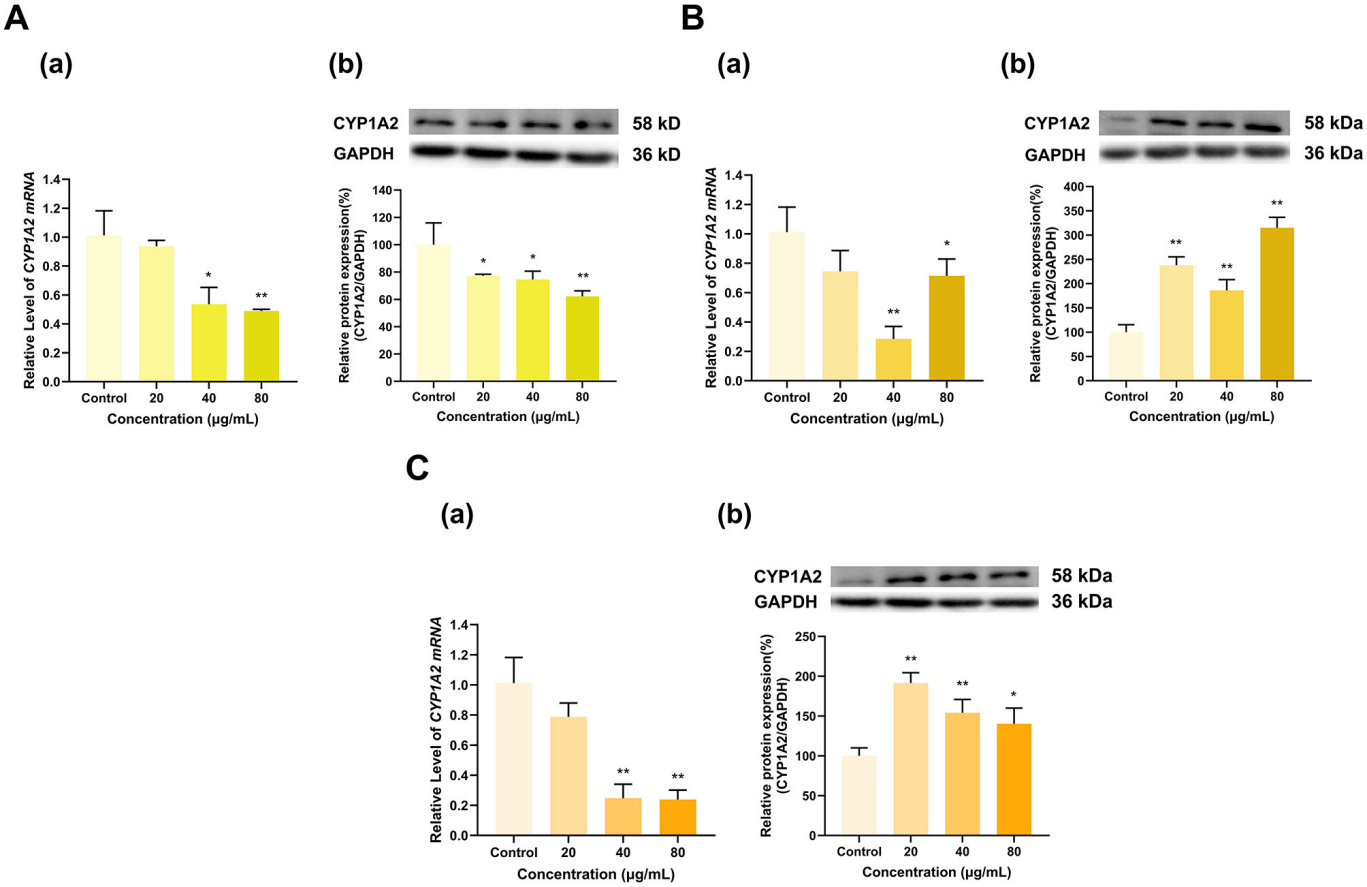
Effect of Theaflavins on CYP1A2 expression in HepG2 cells. **(A)** Effects of TF on the mRNA (a) and protein (b) levels of CYP1A2; **(B)** Effects of TF-3-G on the mRNA (a) and protein (b) levels of CYP1A2; **(C)** Effects of TF-3′-G on the mRNA (a) and protein (b) levels of CYP1A2; The normalized expression data are presented as the mean ± SD (*n* = 3). *p < 0.05 vs. control group, **p < 0.01 vs. control group.

TF, TF-3-G and TF-3′-G significantly affected CYP1A2 expression (Figure 13). TF at 40 and 80 μg/ml decreased the mRNA expression of CYP1A2 to 54% and 49% of that in the control group (*p* < 0.05, *p* < 0.01), respectively, whereas 20 μg/ml TF had no significant effect. At 20, 40, and 80 μg/ml, TF decreased CYP1A2 protein expression to 77%, 75%, and 62% of that in the control group (*p* < 0.05, *p* < 0.05, *p* < 0.01), respectively (Figure 13A). TF-3-G and TF-3′-G inhibited CYP1A2 mRNA expression and induced CYP1A2 protein expression. TF-3-G at 40 and 80 μg/ml decreased CYP1A2 mRNA expression to 28% and 71% of that in the control group (*p* < 0.01, *p* < 0.05), respectively, and had no significant effect at 20 μg/ml. At 20, 40, and 80 μg/ml, TF-3-G increased CYP1A2 protein expression to 238%, 186% and 315% of that in the control group, respectively (*p* < 0.01 for all) (Figure 13B). Similarly, TF-3′-G at 40 and 80 μg/ml decreased the expression of CYP1A2 mRNA to 25% and 24% of that in the control group, respectively (*p* < 0.01 for all), and had no significant effect at 20 μg/ml. At 20, 40, and 80 μg/ml, TF-3′-G increased CYP1A2 protein expression to 192%, 154% and 140% of that in the control group (*p* < 0.01, *p* < 0.01, *p* < 0.05), respectively (Figure 13C).

## 4 Discussion

This study focused on the quantitative and qualitative effects of the polyphenol extracts from three teas (Longjing green tea, Tieguanyin oolong tea, and Dianhong black tea) with different degrees of fermentation, as well as their major monomeric components, i.e., catechins (EGCG, EGC, and ECG) and theaflavins (TF, TF-3-G, and TF-3′-G), on the transcription and protein expression levels of the four major CYP450 isoforms (CYP3A4, CYP2E1, CYP2C9, and CYP1A2) and investigated the regulatory mechanisms of tea polyphenols on Cyp450s to investigate the traditional belief that tea may interfere with drug therapy. The effects of tea polyphenols on CYP450 expression in HepG2 cells are summarized in Table 3.

**Table 3:** Effects of tea polyphenols on CYP450 expression in HepG2 cells.

**Enzymes**		**Effects of tea polyphenols on CYP450 expression**								
		**Longjing green tea**	**Tieguanyin oolong tea**	**Dianhong black tea**	**EGCG**	**EGC**	**ECG**	**TF**	**TF-3-G**	**TF-3**′**-G**
CYP3A4	mRNA	Induction	Induction	Induction	Induction	Inhibition	Inhibition	Suppression to induction	Induction	Induction
	protein	Inhibition	Inhibition	inhibition	Induction to inhibition	Inhibition	Inhibition	Inhibition	Induction	Inhibition
CYP2C9	mRNA	Inhibition	Inhibition	Inhibition	Inhibition	Inhibition	Inhibition	Induction	Overall Induction	Induction to inhibition
	protein	Inhibition	Inhibition	Inhibition	Inhibition	Suppression to induction	Induction to inhibition	Suppression to induction	Inhibition	Inhibition
CYP2E1	mRNA	Inhibition	Inhibition	Inhibition	Inhibition	Inhibition	Inhibition	Induction	Inhibition	inhibition
	protein	Inhibition	Inhibition	Inhibition	Inhibition	Inhibition	Inhibition	inhibition	Inhibition	Induction
CYP1A2	mRNA	Inhibition	Inhibition	Inhibition	Inhibition	Inhibition	Inhibition	Inhibition	Inhibition	inhibition
	protein	Inhibition	Inhibition	Inhibition	Inhibition	Induction	Induction	Inhibition	Induction	Induction

Tea, a beverage native to China, ranks as the second favorite drink in the world, with different flavors and effects depending on the degree of fermentation (8). Therefore, three representative teas, Longjing green tea (unfermented), Tieguanyin oolong tea (semifermented) and Dianhong black tea (fully fermented) were selected as the research objects in this study. Tea contains more than 30 types of tea polyphenols, accounting for 15% to 30% of its dry weight, and these compounds are key to its main biological and pharmacological functions (25). This study investigated tea polyphenol extracts to thoroughly assess the combined effects of various components on CYP activity. Although tea is widely consumed, there is limited information on its safety and interactions with drugs. Tea polyphenols are metabolized mainly by CYP450s, and CYP3A4, CYP2E1, CYP2C9 and CYP1A2, as the most important phase I metabolic enzymes in the CYP450 family, are involved in the biotransformation of various endogenous and exogenous substances in the body (26). In addition, substances that are metabolized by CYP450s can also act on CYP450s, and tea polyphenols influence CYP enzymatic activities by interacting with particular sites on CYP molecules (27).

Enzyme inhibition occurs more frequently than induction, with approximately 70% and 23% of metabolic interactions being due to inhibition and induction, respectively (28, 29). In our study, Longjing, Tieguanyin and Dian Hong tea polyphenol extracts all had inhibitory effects on CYP2E1, CYP2C9 and CYP1A2, indicating that the three tea polyphenol extracts could restrain their enzymatic activities. This may result in increased drug exposure, which could cause adverse reactions or decrease drug effectiveness. In addition, the three tea polyphenol extracts significantly induced CYP3A4 mRNA expression but significantly inhibited its protein expression. Posttranslational regulation or modifications, such as phosphorylation, glycosylation, or ubiquitination, delayed effects, posttranslational stability and regulation by negative feedback mechanisms have been reported to cause inconsistent expression at the mRNA and protein levels (30–32). Our previous animal experiments revealed that black tea polyphenol extracts inhibited the expression of CYP1A2, CYP2E1, and CYP2C37 but induced the expression of CYP3A11 (33). This is in general agreement with the results of the present study. Similarly, Albassam (34) reported that the activities of CYP3A4, CYP1A1, CYP1A2, CYP2C8, CYP2C9, CYP2D6 and CYP2B6 were inhibited by green tea extract and its primary catechin, EGCG, to different extents.

According including EGCG, EGC, and ECG, were the most dominant compounds in the polyphenol extracts of Longjing green tea, Tieguanyin oolong tea and Dianhong black tea. In addition, the Dianhong black tea polyphenol extract was rich in theaflavins, mainly TF, TF-3-G, and TF-3′-G. Therefore, we incubated human HepG2 cells with the six major catechin and theaflavin monomers (EGCG, EGC, ECG, TF, TF-3-G, and TF-3′-G) to explore their mechanisms of CYP450 regulation. The study revealed that EGCG, EGC, and ECG induced the expression of CYP3A4, and although their effects on the expression of CYP2E1, CYP2C9, and CYP1A2 were inhibitory, the exact effects differed. This finding is essentially consistent with the effects of the polyphenol extracts of Longjing green tea, Tieguanyin oolong tea and Dianhong black tea on CYP450, suggesting that EGCG, EGC, and ECG are the major active components of the tea polyphenol extracts. In addition, theaflavins (TF, TF-3-G, and TF-3′-G), the main components of Dian Hong tea, exhibited significant regulation of both CYP450 mRNA and protein expression.

The effects of tea polyphenols on CYP450 vary and can be inconsistent or even contradictory, depending on experimental conditions such as *in vivo* or *in vitro* setting, administered dose, tea type, treatment, and assay method. Wang et al. (35) applied a probe cocktail along with HPLC–tandem mass spectrometry (MS/MS) to evaluate the influence of tea on CYP450 activity, and the results revealed that green tea, black tea, and Tieguanyin tea inhibited CYP1A2 activity to different degrees, whereas Pu'er tea did not have a significant effect. Additionally, the four teas examined did not have a significant effect on the activity of CYP2D6, CYP2C19, or CYP3A1/2. Predicting *in vivo* effects is complicated owing to interactions among different dietary components, individual metabolic abilities, and CYP polymorphism among individuals. Zhang and Qiu (36, 37) reported that Baiyedancong Oolong tea, EGCG and CAF notably elevated the mRNA levels of CYP3A11 and CYP2C37 in the livers of mice but decreased the transcription of CYP2E1; however, different observations have also been reported. According to a preclinical study conducted by Chow et al. (38), four weeks of green tea catechin treatment did not change the phenotypic indices of CYP1A2, CYP2D6, or CYP2C9 and only weakly inhibited CYP3A4 activity. The clinical significance of flavonoid-induced CYP3A4 inhibition in dietary settings is typically regarded as low because of intricate interactions and lower concentrations of individual compounds, but high-dose supplementation or simultaneous use with drugs processed by CYP3A4 necessitates cautious evaluation to prevent potential harmful interactions (39). Hegazy (40) explored how sildenafil and green tea interact in healthy male volunteers and reported that green tea increased the bioavailability of sildenafil by ~50% by inhibiting CYP3A4 expression in the small intestine. Thus, information on CYP450 activity in the literature is inconsistent; furthermore, such discrepancies may be due to the stability and bioavailability of tea polyphenols as well as the duration of action in the experiments. Our experimental results indicate that the three tea polyphenol extracts and their main monomers have different effects on the expression of CYP450s, with an overall induction effect on CYP3A4 and an overall inhibitory effect on CYP2E1, CYP2C37, and CYP1A2. Further investigations into tea polyphenols should aim to determine safe daily doses for humans, with a focus on interactions at the biotransformation stage.

Guvench (41) proposed that water exchange from the buried binding sites of CYP1A2, CYP2D6, and CYP3A4 is linked to conformational changes. Kampschulte et al. (42) reported that dietary polyphenols, particularly flavonoids and stilbenoids, inhibit the CYP450 monooxygenase branch of the arachidonic acid cascade with significant structure-dependent selectivity and potency. However, additional studies are needed to gain a clearer understanding of how polyphenols affect human health through this newly identified molecular mechanism. In addition, various factors can influence CYP450 expression, such as those affecting the attachment of a nuclear receptor to CYP450, competitive inhibition due to the direct binding of a substance to the enzyme, or metabolic influences (43). Studies have shown that nuclear receptors are key transcription factors that regulate CYP450 gene expression and that the inhibitory or induction effects of endogenous and exogenous substances on CYP450 enzyme activity often result from their activation of multiple nuclear receptors as ligands (44). Our earlier research indicated that tea polyphenol extracts increased the transcriptional activation of the CYP3A4 promoter through PXR (33). Furthermore, the PXR–CYP450 pathway does not operate in isolation. The regulatory region of CYP450 attaches to different nuclear receptors, impacting gene expression.

Polyphenolic compounds can influence the pharmacokinetics of corresponding drugs by altering CYP450 activity, particularly affecting drugs primarily metabolized by CYP450 (such as sildenafil and statins) (45, 46). Such changes directly impact clinical efficacy and may even trigger adverse reactions (47). For example, the combined use of tea polyphenols and statins increases the risk of statin-induced myopathy (48). Furthermore, extensive research indicates that green tea consumption significantly reduces systemic drug exposure for multiple medications (including folic acid, lisinopril, celiprolol, digoxin, rosuvastatin, nadolol, nintedanib, atorvastatin, raloxifene, and fexofenadine), with reductions ranging from 18% to 99%, and green tea catechins are the primary contributors (49, 50). Additionally, dietary polyphenols have been reported to prolong the *in vitro* half-life of palbociclib and ribociclib by 6.4-fold, with the potential mechanism linked to dietary polyphenols acting as CYP3A4 inhibitors (51).

## 5 Conclusion

Tea, which is a commonly consumed beverage and dietary supplement, can significantly reduce the risk of many diseases. In this study, we systematically evaluated the effects of three tea polyphenol extracts (those of Longjing green tea, Tieguanyin oolong tea, and Dianhong black tea) and their major constituent monomers, i.e., catechins (EGCG, EGC, and ECG) and theaflavins (TF, TF-3-G, TF-3′-G), on CYP450s. Our results show that the tea polyphenols significantly affected CYP450 expression and that enzyme inhibition was more prevalent than enzyme induction, with large contributions from the major monomers. HepG2 cells have limited metabolic capacity compared to primary human hepatocytes and that results may not fully represent *in vivo* human metabolism. Comprehensive consideration should be taken in subsequent research, including studies on primary human hepatocytes, nuclear receptors such as PXR and CAR, drug transporter proteins such as P-glycoprotein (P-gp), and multidrug resistance-associated protein (MRP) families, to better evaluate the potential influence of tea polyphenols on the effects of drugs and provide a scientific basis for tea consumption during drug therapy. In addition, neither animal nor cellular experiments can replace clinical trials, and we plan to verify the effects of tea on drug action through clinical trials in the future.

## Data Availability

The original contributions presented in the study are included in the article/supplementary material, further inquiries can be directed to the corresponding author.
